# Mechanisms of microbe-mediated immune development in the context of antibiotics and asthma

**DOI:** 10.3389/falgy.2024.1469426

**Published:** 2024-10-14

**Authors:** Katherine Donald, B. Brett Finlay

**Affiliations:** ^1^ Michael Smith Laboratories, University of British Columbia, Vancouver, BC, Canada; ^2^ Department of Microbiology and Immunology, University of British Columbia, Vancouver, BC, Canada; ^3^ Department of Biochemistry and Molecular Biology, University of British Columbia, Vancouver, BC, Canada

**Keywords:** asthma, allergies, microbial metabolites, microbe-mediated immune imprinting, antibiotics

## Abstract

The gut houses 70%–80% of the body's immune cells and represents the main point of contact between the immune system and the outside world. Immune maturation occurs largely after birth and is guided by the gut microbiota. In addition to the many human clinical studies that have identified relationships between gut microbiota composition and disease outcomes, experimental research has demonstrated a plethora of mechanisms by which specific microbes and microbial metabolites train the developing immune system. The healthy maturation of the gut microbiota has been well-characterized and discreet stages marked by changes in abundance of specific microbes have been identified. Building on Chapter 8, which discusses experimental models used to study the relationship between the gut microbiota and asthma, the present review aims to dive deeper into the specific microbes and metabolites that drive key processes in immune development. The implications of microbiota maturation patterns in the context of asthma and allergies, as well as the effects of antibiotics on microbe-immune crosstalk, will also be discussed.

## 1 Introduction

### 1.1 The infant gut microbiota: general patterns in colonization

The gut microbiota is highly dynamic during the neonatal period, and does not reach stability until 3–5 years of age (1). Although there is a basic trajectory of colonization common to most infants, numerous environmental and host factors shape the progression of microbiota establishment. At birth, the infant gut is colonized by aerobic and facultative anaerobic bacteria largely belonging to the Proteobacteria phylum, including *Enterobacteriaceae* species such as *Escherichia coli* and *Klebsiella*. These microbes consume oxygen and establish an anaerobic niche within the gut, enabling colonization of primarily Actinobacteria (including *Bifidobacteria* species), along with members of the Firmicutes and Bacteroides phyla over the first months of life (2, 3). Birth mode is the major determinant of microbiota composition in the first weeks of life (4).

The “Bifidobacterium peak”, a period during which *Bifidobacteria* species dominate in the infant gut, is established as aerobic bacteria rapidly drop in numbers, and persists for the first few months of life. Feeding practice replaces birth mode as the major determinant of gut microbiota composition. Breastmilk contains human milk oligosaccharides (HMOs), complex prebiotic sugars which directly promote *Bifidobacteria* species and cannot be digested by the infant (5). Breastfed infants thus display a stronger and more persistent Bifidobacterium peak than formula-fed infants. Around the time of solid food introduction (4–6 months), Bifidobacteria species decline and are replaced gradually by Clostridia and some Bacteroides species (6, 7).

The maturation patterns of the infant gut microbiota have been well-characterized through mathematical models, which are designed predict age based on gut microbiota composition (8, 9). Using these models, researchers have found that slow diversification and a strong and persistent Bifidobacterium peak are the hallmarks of healthy microbiota maturation, and that premature diversification is associated with poor health outcomes (7, 10).

### 1.2 Infant immune development: key features

**Figure 1:**
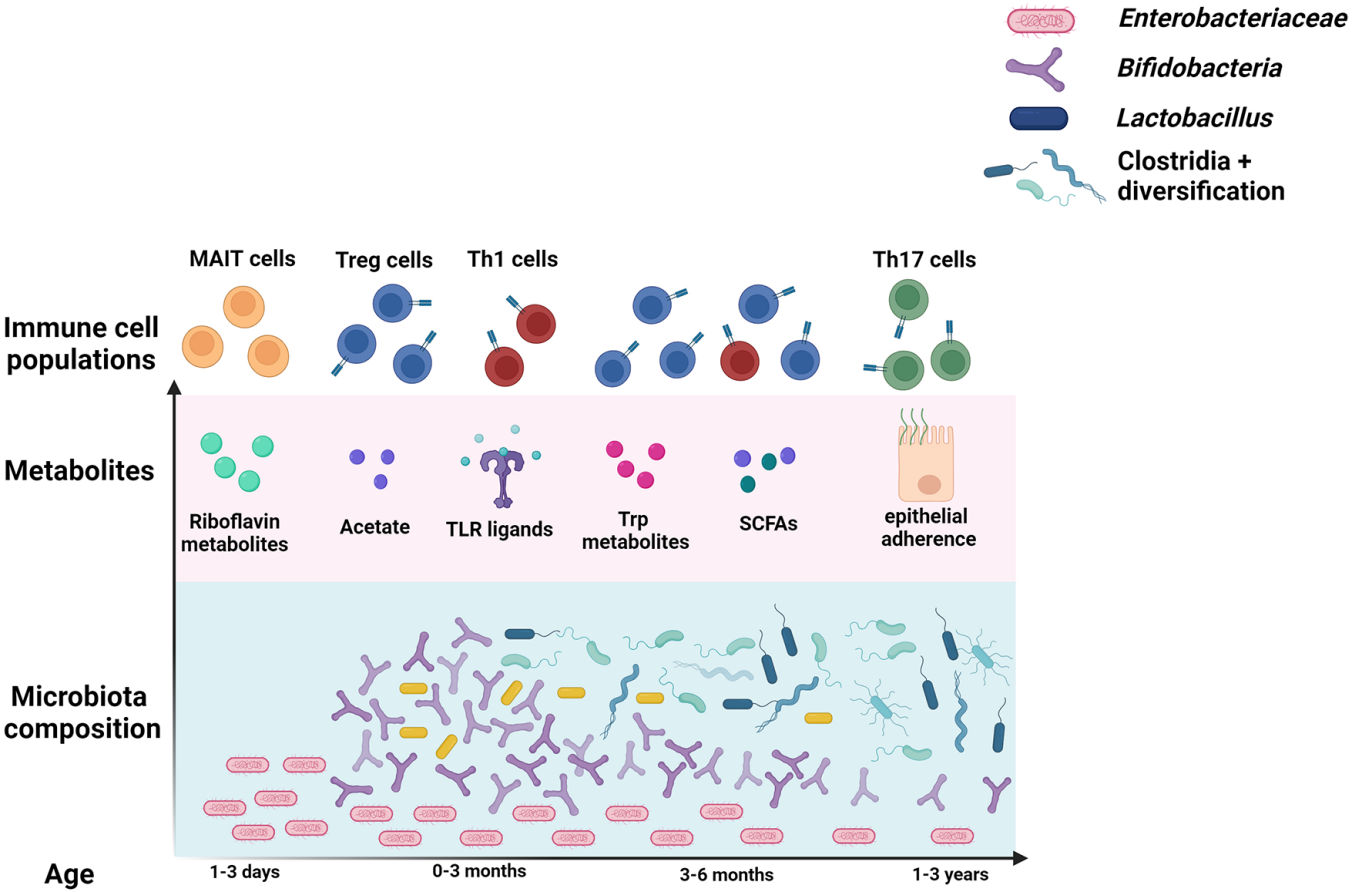
Microbe-mediated immune system imprinting in early life.

The period between birth and 3–5 years of age represents a critical period of microbe-mediated immune imprinting that affect life-long systemic health (Figure 1) (11, 12). However, some compartments of the immune system develop *in utero*, and may be affected by the maternal microbiota. Although the placenta is devoid of bacteria, cytokines and bacterial metabolites can cross the placenta, enabling cross-talk between maternal immune responses to the gut microbiota and fetal development (13). De Agüero et al. were able to transiently colonize otherwise germ-free pregnant mice with *Escherichia coli*, such that pups would only experience the effects of the microbe *in utero* (14). They found that pups born to transiently colonized dams displayed increases in several immune compartments important for recognizing and responding to the colonizing microbiota, and that transfer of maternal antibodies across the placenta was responsible for this. This indicates that the influence of the microbiota on immune maturation begins even before birth.

The neonatal immune system is uniquely suited to face the influx of antigenic stimulation that occurs at birth. Newborn T cell responses that are prone toward regulatory and Th2 phenotypes, limiting Th1-mediated inflammation that would typically be induced by foreign bacterial antigens (15). Newborns also display reduced blood neutrophil and monocyte levels, and impaired Toll-like receptor-mediated microbial recognition (16–18). Regulatory cytokines IL-10 and IL-27, which limit inflammation, are elevated in infant blood. Although B cell and dendritic cell (DC) responses converge with maternal phenotypes by 3 months, T cell responses take longer to fully develop, and inflammatory Th1 cell levels increase only after 1 month of age (19). These results come from studies of human peripheral and cord blood samples taken in the first moments or days of life, and illustrate that neonatal systemic immune system is poised to tolerate the colonization and establishment of the gut microbiota.

The gut harbors 70%–80% of the body's immune cells, and analyses of human blood fail to capture immune development occurring at this important mucosal barrier (20). Due to the difficulty in obtaining intestinal tissue samples from human infants, most of our knowledge of gut mucosal immune development comes from animal studies. While the specific timing and order of immune maturation differ between species, the role of microbes and the general patterns of gut development are similar. In both mouse and human neonates, mucous secretion, cell proliferation, antimicrobial peptide production, and immune cell numbers are reduced (21). Over the course of the first few weeks in mice and months in humans, microbial expansion drives the development of an intact mucosal barrier. The interactions that occur during this period not only affect local processes such as oral tolerance and defense against intestinal pathogens and pathobionts, but also systemic immunity and allergy susceptibility (22). The following sections will highlight the major microbes and metabolites that affect specific immune cell populations at both the mucosal and systemic level, with a focus specifically on T cell populations that affect asthma and allergy outcomes. The progression of infant gut microbiota and T cell development is summarized in Figure 1.

## 2 Short chain fatty acids

Short chain fatty acids (SCFAs) are the most well-characterized bacterial products that affect systemic immunity. Acetate, propionate, and butyrate, the most common SCFAs, are produced in the breakdown of prebiotics such as dietary fiber (23). They are taken up by colonocytes and released into circulation to affect a variety of cell types. They primarily bind to either G protein coupled receptors, affecting intracellular pathways, or histone deacetylases, turning on gene expression (24). Among countless other functions throughout the body, SCFAs promote regulatory and anti-inflammatory immune responses.

Acetate alone rescues the altered thymic T cell development observed in germ-free mice (25), and is anti-inflammatory in cultured human-derived organoids (26). All 3 major SCFAs can promote IL-10 production and regulatory T cell (Treg) phenotypes, contributing to immune tolerance and limiting inflammation. This is achieved through direct binding to G protein coupled receptors, affecting downstream pathways in immune cells or by direct inhibition of histone deacetylases that act on *Foxp3,* the gene that encodes the Treg defining transcription factor in the gut (27). Butyrate can also promote gut barrier integrity and limit gut permeability by inducing tight junction expression and mucous production (28).

During the first few months of life, *Bifidobacteria* species, which make up the majority of the gut microbiota in breastfed infants, produce high levels of acetate in the breakdown of HMOs (Figure 1) (29). Changes in diet and environmental exposure, along with cross-feeding of acetate, promote the slow rise of butyrate and propionate producing bacteria mainly belonging to the Firmicutes phylum. While some *Prevotellaceae* species in the Bacteroides phylum produce SCFAs, the *Lachnospiraceae* family includes the most butyrate and propionate producers (30). Dietary fiber replaces HMOs as the primary substrate for SCFA production, contributing to a stable and healthy gut community.

The anti-inflammatory effects of SCFAs have been shown to directly limit allergic phenotypes in animal studies. Feeding mice a high fiber diet promotes SCFA producers and limits inflammatory responses in asthma models by increasing Treg responses (31, 32). The weaning reaction to the colonizing microbiota, discussed in Chapter 8, involves SCFA-induced Treg development which protects against later development of Th2-mediated disease (33). Importantly, microbial exposure after weaning is not sufficient to rescue the phenotype, implicating the early-life window as an essential period of SCFA-mediated immune imprinting. SCFAs can even cross the placenta, and acetate has been shown to promote Treg development in the fetal lung and protect against later development of asthma when produced at high levels by the maternal microbiota (34).

The role of SCFAs in allergy protection is also supported by human data. Levels of acetate-producing Bifidobacteria before 6 months are inversely correlated with allergic outcomes (35–37). The abundance of several SCFA producing *Lachnospiraceae* species are also thought to be protective against allergies (38). Levels of butyrate producers are reduced in 1 year old infants that develop allergies (37), and plasma levels of SCFAs are reduced in infants who go on to develop atopic disease (39). Additionally, infants born to mothers who carry the acetate-producer *Prevotella copri* during pregnancy were found to be significantly less likely to develop food allergies (40). This relationship did not depend on whether the infant also carried *P. copri*, potentially implicating trans-placental acetate in the protective effect.

## 3 Microbial stimulation of regulatory pathways

SCFAs are not the only anti-inflammatory metabolites produced by bacteria in the gut. Indole-3-lactic acid, produced by *Bifidobacterium infantis* in tryptophan catabolism*,* promotes expression of the negative regulator galectin-1 in Th2 and Th17 cells, contributing to tolerance and limiting inflammation in human infants (41). This was cleverly demonstrated by exposing naïve CD4+ T cells to fecal water extracted from infants supplemented with a *B. infantis* probiotic. Other tryptophan metabolites, produced by a variety of bacteria, including *Lactobacillus reuteri,* also promote Treg responses by binding to Aryl Hydrocarbon receptors (AhR) expressed by intestinal epithelial and immune cells (42, 43).

As mentioned above, the neonatal immune system is alternatively programmed compared to that of adults. The alterations have been described in detail previously, but can be generally summarized as a proneness toward regulatory responses (7). Thus, some antigens that typically induce inflammation actually drive anti-inflammatory responses in infants. For example, newborn DCs produce the regulatory cytokine IL10 in response to the endotoxin LPS (16). Between shifts in the microbiota and the altered immune state of infants, there are numerous mechanisms in place to prioritize Treg development, and for good evolutionary reason: Treg levels seem to be imprinted in early life, as indicated by the “weaning reaction” and the fact that germ-free mice lack intestinal Tregs, a phenotype that can only be restored by colonization during the neonatal period (44). Therefore, sufficient host-microbe interactions specifically during the first months of life are vital to developing tolerance and a strong Treg pool.

## 4 Microbial stimulation of inflammatory pathways

One of the original theories to explain the hygiene hypothesis was the Th1/Th2 paradigm: the idea that Th1 cells and Th2 cells reciprocally regulate each other, and that sufficient stimulation of Th1 responses was required to dampen Th2 responses involved in allergy (45, 46). Although cytokines produced by Th1 and Th2 cells do limit one another, T cells display a high level of plasticity, and alternative T cell types have emerged. Thus, the idea that the adaptive immune system exists simply as a “balance” between Th1 and Th2 cells has been largely debunked (47). However, there is ample evidence that in addition to promoting regulatory and anti-inflammatory responses, the gut microbiota must be sufficiently diverse and immunostimulatory in order to favor non-Th2 responses and promote proper immune development.

Endotoxin, a pro-inflammatory molecule released by gram-negative bacteria, is a potent stimulator of Th1 responses. In addition to promoting Treg responses, as mentioned above, endotoxin contributes to a slow maturation of Th1 responses (48). As the gut epithelium matures and becomes more proliferative, intracellular endotoxin levels shift, which slowly trains Th1 cells to respond appropriately. Oral endotoxin was also shown to dampen Th2-mediated inflammation in a murine asthma model, likely by limiting Th2-skewed DC recruitment to the lung (49, 50). Polysaccharide A (PSA), produced by *Bacteroides fragilis,* is taken up by intestinal DCs and carried to lymphoid organs to induce Th1 cells, limiting Th2 inflammation (51). Strains of *Lactobacillus*, commonly implemented as probiotics, also promote Th1-inducing cytokines by binding and stimulating innate Toll-like receptors expressed by intestinal epithelial cells (52). This has been shown to ameliorate inflammation in the OVA model of allergic asthma (53).

More recently, Th17 cells have emerged as an abundant and important T cell type particularly in the gut. Th17 cells promote barrier integrity, mucous production, and pathogen responses (54). These cells are completely lacking in GF mice, a phenotype that can be rescued by transplantation of a diverse microbiota or colonization with specific tissue-adherent microbes (44). Th17 cell responses in humans are also likely stimulated by microbes that adhere closely to the epithelial lining, rather than production of a specific metabolite (55). Although Th17 cells are protective in the context of pathogens and oral tolerance, overstimulation of Th17 pathways can contribute to allergic asthma phenotypes in humans and mouse models (56–58). A balance between Th17 and Treg pathways is thus essential for protecting against both pathogens and allergies.

Mucosal-associated invariant T (MAIT) cells, a type of innate-like lymphocyte that develop during the neonatal period, have also recently been recognized as important players in the microbiota-immune axis and asthma. Similar to Th17 cells, MAIT cells contribute to inflammation and pathogen resistance, but are thought to actually limit Th2 responses and asthma (59, 60). MAIT cells are missing in GF mice, a phenotype that can only be rescued during early life by colonization with *Enterobacteriaceae* capable of metabolizing riboflavin into MAIT-inducing antigens (61). As mentioned above, *Enterobacteriaceae* are the first colonizers of human infants, and the relationship between this family and MAIT cells may provide an evolutionary reason for this (62).

## 5 Antibiotics and mechanisms of microbe-immune crosstalk

There is extensive evidence that a loss of SCFAs and Tregs partially mediate the detrimental immunological effects of early life antibiotic exposure. In mice, vancomycin-induced dysbiosis in early life led to a reduction in butyrate-producing Clostridia and more severe allergic lung inflammation (63, 64). SCFA supplementation was sufficient to rescue the antibiotics-associated allergic phenotype. Cefoperazone treatment also significantly reduced SCFA levels in mice (65). Antibiotics also completely disrupt the weaning reaction, permanently reducing the levels of allergy-protective Tregs (33). In humans, antibiotic treatment disrupts the Bifidobacterium peak permanently, and transiently alters *Lachnospiraceae* levels, both of which are important contributors of SCFAs in infancy (66).

In addition to weakening the Bifidobacterium peak, antibiotics limit Bacteroides species and overall diversity in infants, which could limit Th1 induction by the microbiota and contribute to Th2-skewing and allergies (67). Recovery from antibiotics involves an early emergence of Clostridia species, and a faster diversification of the gut microbiota. While Clostridia have beneficial roles in immune activation, through SCFA production and Treg induction, some members of this class promote Th17 responses (66). Therefore, premature Clostridia colonization and general diversification in replacement of the Bifidobacterium peak may promote early and elevated Th17 responses and limit Treg induction by *Bifidobacteria*, contributing to allergic disease susceptibility. Antibiotics have also been shown to limit MAIT cell development by targeting riboflavin metabolizing microbes (68). As expected, this altered phenotype was specific to antibiotic exposure during the early-life period.

## 6 Conculsions

As communicated in this review, early life is a key period of immune maturation that is guided by the gut microbiota. The immune system of infants is uniquely prepared to face and respond to microbial stimulation, and the timing of antigenic exposure shapes the delicate balance of immune populations that affect life-long systemic health. There are several key stages of gut microbiota maturation that occur alongside mucosal and immune development: Initial colonization with *Enterobacteriaceae* species contributes to MAIT cell development, a strong and prolonged “Bifidobacterium” peak drives Treg development through SCFAs and other metabolites, and a slow diversification and emergence of new SCFA producers promote barrier integrity, appropriately trained Th1 responses, and continued tolerance. Antibiotic administration during infancy significantly disrupts many of these key processes, and the studies described above demonstrate some of the many mechanistic explanations for the long-term detrimental effects of antibiotics on host health and allergic disease susceptibility.

Microbiota composition is summarized over the course of the first few years of life in blue. *Enterobcateriaceae* species seed the intestine first. The “bifidobacteria peak” occurs shortly after, and is defined by the breastfeeding period. Lactobacillus are also more abundant during the first months of life. Clostridia levels rise next, and overall diversity increases over the course of the first few years of life. The most well-studied metabolites produced by these microbes, and the T cell types they primarily stimulate, are displayed above the microbes that generally produce them.
